# Glymphatic system dysfunction in chronic tinnitus patients with sleep disturbance

**DOI:** 10.3389/fneur.2025.1504645

**Published:** 2025-06-10

**Authors:** Yinjuan Du, Zhichun Huang, Jin-Jing Xu, Yuan Xue, Zigang Che

**Affiliations:** ^1^Department of Otolaryngology-Head and Neck Surgery, Zhongda Hospital, Southeast University, Nanjing, China; ^2^Department of Otolaryngology, Nanjing First Hospital, Nanjing Medical University, Nanjing, China; ^3^Department of Otolaryngology, Nanjing Pukou People's Hospital, Nanjing, China; ^4^Department of Radiology, Nanjing Tongren Hospital, School of Medicine, Southeast University, Nanjing, China

**Keywords:** chronic tinnitus, sleep disturbance, glymphatic system, magnetic resonance imaging, diffusion tensor imaging

## Abstract

**Purpose:**

The neural mechanisms of sleep disturbance associated with chronic tinnitus remains unknown. To investigate this issue, multimodal magnetic resonance imaging (MRI) was used to detect glymphatic system dysfunctions in chronic tinnitus patients with sleep disturbance.

**Methods:**

This prospective study included 30 tinnitus with sleep disturbance (TSD), 30 tinnitus with no sleep disturbance (TNSD) and 38 age, sex, and education-matched healthy controls (HCs). All the subjects underwent MRI scans of the glymphatic indexes and clinical assessment. Multimodal MRI indices were used as proxies of glymphatic function and the relationships between the glymphatic function and sleep disturbance were further evaluated.

**Results:**

TSD group exhibited significantly higher choroid plexus volume (CPV) and enlarged perivascular spaces (EPVS) values than the HCs group (*p* < 0.0001). Moreover, the TNSD group revealed significantly lower diffusion tensor image analysis along the perivascular space (DTI-ALPS) values than the HCs group (*p* = 0.044). In chronic tinnitus patients, the decreased DTI-ALPS index was negatively associated with the Pittsburgh Sleep Quality Index (PSQI) scores (*r* = −0.428, *p* = 0.001). In addition, the increased CPV and EPVS values were positively correlated with the PSQI scores (*r* = 0.374, *p* = 0.005; *r* = 0.335, *p* = 0.013; respectively). Furthermore, reduced ALPS values were negatively associated with the Tinnitus Handicap Questionnaires (THQ) scores (*r* = −0.378, *p* = 0.005).

**Conclusion:**

Using multimodal MRI approaches, this study provides preliminary evidence for disrupted glymphatic function in chronic tinnitus patients, which may be associated with sleep disturbance. CPV, EPVS, and ALPS could serve as neuroimaging markers and shed new light on neuropathological mechanisms for chronic tinnitus comorbid with sleep disturbance.

## 1 Introduction

Chronic tinnitus is the permanent perception of a sound with no identifiable corresponding acoustic source (1–3). Tinnitus patients often suffer from sleep problem and psychological distress such as depression and anxiety that significantly influence the life quality (4–6). The prevalence of comorbid sleep disturbance in tinnitus patients ranged from 10.1% to 79.5% (7). Previous researches have elaborated on the relationship between chronic tinnitus and sleep disturbance (8–10). However, the neurophysiological mechanism of sleep disturbance associated with tinnitus remains unknown.

Recent studies have shown that the glymphatic system plays a pivotal role in removing metabolic waste from the brain (11). The glymphatic system involves the interaction between cerebrospinal fluid (CSF) and brain interstitial fluid (ISF). The fluid then exits the brain parenchyma through venous perivascular spaces, clearing waste such as amyloid beta (Aβ) and tau protein into meningeal lymphatic vessels (12). When the clearance function for Aβ and tau protein is compromised, the balance will be broken, and the accumulation and aberrant deposition of Aβ and tau will lead to a cascade of damages, ultimately resulting in cognitive decline. Dysfunction in the glymphatic system has been hypothesized to play a role in sleep disorders (11, 13, 14). However, none of these studies have focused on investigating brain glymphatic dysfunction in chronic tinnitus with sleep disturbance to date.

The glymphatic system can be evaluated by proton emission tomography (PET) (11) or gadolinium-based contrast-enhanced magnetic resonance imaging (MRI) (15); however, several non-invasive MRI indices are useful for indirect glymphatic evaluation, such as choroid plexus volume (CPV) (16), enlarged perivascular spaces (EPVS) (17), diffusion tensor image analysis along the perivascular space (DTI-ALPS) (18–20). The CPV integrates signals from the brain parenchyma with signals from circulating immune cells, and selectively recruits peripheral leukocytes to the brain parenchyma (16). The EPVS is a major indicator of the increase in periarterial space, representing the inflow of CSF into the brain parenchyma (21). In addition, the ALPS index can be used for measuring the spatial diffusion around the deep medullary vein, mainly reflecting the outflow of CSF/ISF (22). Therefore, aberrant glymphatic system function purportedly contributes to pathophysiology of brain aging, neurodegenerative diseases, and other brain injuries (23). Our prior study investigated for the first time that lower DTI-ALPS index values were detected in chronic tinnitus patients, which was significantly correlated with lower scores on specific cognitive performance (24). Nevertheless, it is unclear whether the other indices of the glymphatic system were abnormal in chronic tinnitus and if sleep disturbance contributed to the glymphatic system dysfunction associated with chronic tinnitus.

To determine if glymphatic system dysfunction existed in chronic tinnitus patients with sleep disturbance, we used MRI to obtain CPV, EPVS, and DTI-ALPS values of glymphatic function and determined their relationships with sleep disturbance in chronic tinnitus patients. We hypothesized that: (a) CPV, EPVS and DTI-ALPS indices of glymphatic function would be disrupted in chronic tinnitus patients compared to normal controls, (b) that CPV, EPVS and DTI-ALPS values of glymphatic function would be correlated with sleep disturbance in chronic tinnitus patients.

## 2 Materials and methods

### 2.1 Subjects

The current study was approved by the Ethics Committee of Nanjing First Hospital. All subjects provided written informed consent before their participation in this study. Sixty chronic tinnitus patients were included from otolaryngology department while 38 age, sex, and education well-matched HCs were recruited. Pittsburgh Sleep Quality Index (PSQI) was used to evaluate sleep quality of patients with tinnitus. According to the PSQI score, the tinnitus patients were divided into 30 tinnitus with sleep disturbance (TSD) (PSQI score >5) and 30 tinnitus with no sleep disturbance (TNSD) (PSQI score ≤ 5). All individuals were 30–70 years old, right-handed and completed more than 9 years of education. Patients had bilateral or central tinnitus without hearing loss (hearing threshold < 25 dB). The hearing thresholds of both ears were assessed by puretone audiometry (PTA) at the frequencies of 250, 500, 1,000, 2,000, 4,000, and 8,000 Hz. Tinnitus severity was assessed by Tinnitus Handicap Questionnaires (THQ) (25), which was categorized as mild, moderate or severe (26). Ten patients had mild tinnitus, 25 moderate tinnitus, and 25 severe tinnitus. All HCs and most tinnitus patients had normal hearing. Evaluation of tinnitus related depression and anxiety symptoms were assessed using the Self-Rating Depression Scale (SDS) and Self-Rating Anxiety Scale (SAS) (27, 28). Montreal Cognitive Assessment (MoCA) was used to assess the cognitive status for each subject (29). One TSD patient and one TNSD patient were subsequently excluded from the study due to the exceeded limits for head motion during MR scanning.

Exclusion criteria included the following: (1) pulsatile tinnitus, hyperacusis, Meniere's diseases; (2) ear surgery, acoustic neurinoma, use of ototoxic drugs; (3) severe smoking, alcoholism, drug addiction, stroke, head injury, Alzheimer's disease, Parkinson's disease, epilepsy, schizophrenia; (4) other major central nervous system (CNS) disorders; and (5) MRI contraindications. The demographics and clinical information of the chronic tinnitus patients and HCs are presented in Table 1.

**Table 1:** Demographics and clinical information between tinnitus patients and HCs.

**Items**	**TSD (*n =* 29)**	**TNSD (*n =* 29)**	**HCs (*n =* 38)**	***p*-value**
Age (year)	63.62 ± 7.08	62.59 ± 6.85	61.45 ± 3.79	0.330^a^
Gender (male/female)	14/15	13/16	17/21	0.951^b^
Education (years)	10.28 ± 1.67	11.10 ± 1.01	10.63 ± 1.78	0.130^a^
Tinnitus duration (months)	31.86 ± 19.97	24.62 ± 12.25	–	0.787^c^
THQ score	50.69 ± 15.52	51.81 ± 15.92	–	0.102^c^
PTA of left ear (dB HL)	16.72 ± 2.17	16.84 ± 3.06	17.63 ± 4.24	0.482^a^
PTA of right ear (dB HL)	16.49 ± 2.81	17.01 ± 3.13	17.24 ± 3.73	0.654^a^
Average PTA of both ears (dB HL)	16.61 ± 1.99	16.93 ± 2.49	17.43 ± 3.58	0.492^a^
MoCA scores	26.45 ± 0.74	26.66 ± 0.72	26.89 ± 0.86	0.073^a^
PSQI scores	9.59 ± 3.07	4.48 ± 0.63	4.42 ± 0.68	< 0.001^*a^

Data are represented as Mean ± SD.

^*^P < 0.001. ^a^The p-values are obtained by using one-way analysis of variance. ^b^The p-values are obtained by using χ2 test. ^c^The p-values are obtained by using two-sample t-test.

TSD, tinnitus with sleep disturbance; TNSD, tinnitus with no sleep disturbance; HCs, healthy controls; THQ, Tinnitus Handicap Questionnaires; PTA, puretone audiometry; MoCA, Montreal Cognitive Assessment; PSQI, Pittsburgh Sleep Quality Index.

### 2.2 MR data acquisition

MRI data were obtained using a 3.0-T MR imaging system (MAGNETOM Skyra; Siemens Healthcare, Erlangen, Germany) with a 20-channel receiver array head coil. During scanning, earplugs and headphones were used to reduce the scanner noise. The earplugs (Hearos Ultimate Softness Series, USA) were used to attenuate scanner noise by approximately 32 dB. The scan parameters of DTI were as follows: TR = 4,996 ms, TE = 102 ms, slices = 70, slice thickness = 2 mm, gap = 0, FA = 90°, *b*-values = 0 and 1,000s/mm^2^, diffusion gradient directions = 32, matrix = 128 × 128, and FOV = 200 mm × 200 mm. Structural images were obtained using a high-resolution T1-weighted gradient-echo sequence and the following scan parameters: TR/TE = 9.912/4.12 ms, slices = 160, thickness = 1 mm, gap = 0, FA = 16°, matrix = 256 × 256, and FOV = 256 mm × 256 mm.

### 2.3 DTI-ALPS measurement

**Figure 1:**
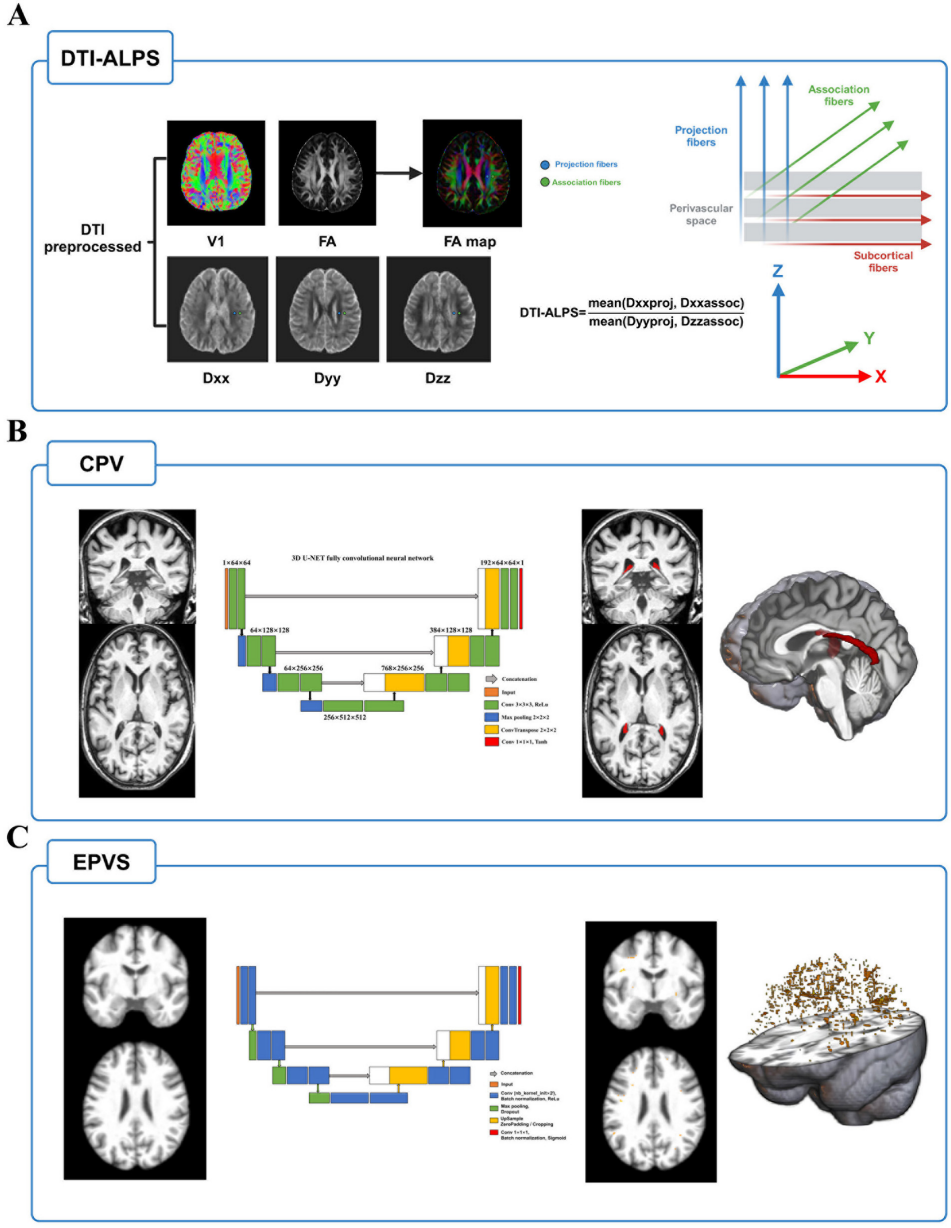
The MRI indices calculation flow of glymphatic system function. **(A)** After preprocessing of DTI, FA and individual diffusivity maps were generated using DTIFIT tool implemented in FMRIB Software Library. Next, placing 5 mm diameter spherical ROIs on the bilateral projection fibers, association fibers, and subcortical fibers and extracting the diffusivities of the three directions along the *x, y*, and *z* axes at the ROIs on bilateral fibers on both sides of the brain. The DTI-ALPS index was calculated based on this formula: mean (Dxxassoc, Dxxproj)/mean (Dzzassoc, Dyyproj). **(B)** Choroid plexus was segmented by inputting T1w image into a validated U-net deep learning model, and its volume was extracted after inspected and reviewed. **(C)** EPVS probability maps were obtained by using another validated U-net deep learning segmentation model. The volume of EPVS is extracted after thresholding and review. Abbreviations: EPVS, enlarged perivascular space; DTI, diffusion tensor image; FA, fractional anisotropy; ROI, regions of interest.

The DTI-ALPS index was calculated from diffusion-weighted imaging data using the DTIFIT tool of the FMRIB Software Library (FSL, Wellcome Centre for Integrative Neuroimaging, University of Oxford, UK, https://fsl.fmrib.ox.ac.uk/fsl/fslwiki/FSL). Detailed calculation process was depicted in Figure 1A and previous study (18). Briefly, preprocessing procedure included format conversion, brain extraction, eddy correction, and tensor calculation firstly. Then, we used fsleyes tool to outline 5 mm diameter spherical regions of interest (ROIs) on the bilateral projection fibers, association fibers, and subcortical fibers. Next, we extracted the diffusivities of the three directions along the x, y, and z axes at the voxel level within the ROI. Finally, the DTI-ALPS index was calculated based on this formula: mean (Dxxassoc, Dxxproj)/mean (Dzzassoc, Dyyproj). All the outlines of ROI were reviewed by an experienced radiologist.

### 2.4 CPV measurement

To reduce the potential error of manual segmentation, we chose a convolutional neural network model using U-Shaped Neural Network (U-NET) architecture to segment the volume of the choroid plexus using T1w MRI (https://github.com/hettk/chp_seg) (30). This architecture had been validated across the adult lifespan and shown better performance than the existing segmentation methods (such as FreeSurfer). More detailed segmentation process was depicted in Figure 1B. Briefly, individual T1w image was used as input and registered non-linearly with Advanced Normalization Tools (ANTs) to the International Consortium for Brain Mapping-Montreal Neurological Institute (ICBM-MNI) 152 -T1-weighted template (31). After processing by the 3D U-NET fully convolutional neural network, the output was then inversely transformed to the native imaging space and generated a mask of choroid plexus. Finally, the volume is calculated using SimpleITK (version 2.1.1.1) in Python (3.7.0) to acquire the individual choroid plexus volume (32). Furthermore, we used Computational Anatomy Toolbox (CAT12, Jena University Hospital, Departments of Psychiatry and Neurology, Germany, https://github.com/ChristianGaser/cat12) in Statistical Parametric Mapping software package (SPM12, Functional Imaging Laboratory, Wellcome Department of Cognitive Neurology, UCL Queen Square Institute of Neurology, UK, http://www.fil.ion.ucl.ac.uk/spm) to segment T1 images and obtained total intracranial volume (TIV), gray matter volume (GMV), white matter volume (WMV), and cerebrospinal fluid volume (CSFV). In addition to the volume raw value, it was expressed as a ratio of TIV ^*^ 1,000 to eliminate potential influences of individual variability in brain volume and further analyses were based on this ratio.

### 2.5 EPVS measurement

EPVS probability map of each T1w image was generated using a previously validated deep learning model (33). This model has been conducted to quantify EPVS in cerebral vessel disease and demonstrated satisfactory effectiveness and robustness (17). Based on the suggestions of the developers and manual review of the EPVS probability map, we adopted a threshold of 0.1 to maximize the inclusion of all potential EPVS and generate individual EPVS masks. Finally, similar to the extraction process of CPV, the original EPVS volume was extracted and was expressed as a ratio of TIV ^*^ 1,000. Detailed segmentation process was depicted in Figure 1C.

### 2.6 Statistical analysis

The differences in demographic and clinical information were investigated using SPSS 26.0 (SPSS, Inc., Chicago, IL, USA). The chi-square test was used for categorical variables such as gender, while the independent samples *t*-test or Mann–Whitney U-test was used for continuous variables with normally distributed data, based on normal distribution tested with the Shapiro–Wilk test. The MRI index was calculated for each group and its correlation with clinical variables was assessed using Pearson's correlation. Bonferroni correction for multiple comparisons was carried out. Statistical significance was determined using a two-tailed *p*-value of < 0.05.

## 3 Results

### 3.1 Demographic and clinical data

The demographic and clinical data from three groups are presented in Table 1. No significant differences were detected among TSD, TNSD patients and HCs in terms of age, gender, education level, average hearing thresholds, and MoCA scores (*p* > 0.05). There were no significant differences of THQ scores and disease duration between TSD and TNSD patients (*p* > 0.05). Compared with HCs and TNSD, TSD patients revealed significantly worse performances on the PSQI scores (*p* < 0.001).

### 3.2. Structural results

Compared with HCs, no significant differences of GMV, WMV, and TIV were detected among tinnitus patients and HCs (*p* > 0.05) (Table 2). After Monte Carlo simulation correction, we detected no suprathreshold voxel-wise differences of GMV, and WMV among tinnitus patients and HCs.

**Table 2:** Comparison of the brain volume and glymphatic function characteristics between tinnitus patients and HCs.

**Items**	**TSD (*n =* 29)**	**TNSD (*n =* 29)**	**HCs (*n =* 38)**	***p*-value**
Total intracranial volume (TIV) (cm^3^)	1343.47 ± 131.10	1352.83 ± 135.14	1349.11 ± 138.27	0.965
Gray matter volume (% of TIV)	0.323 ± 0.023	0.322 ± 0.017	0.325 ± 0.019	0.885
White matter volume (% of TIV)	0.300 ± 0.016	0.294 ± 0.014	0.294 ± 0.016	0.282
Brain parenchyma volume (% of TIV)	0.623 ± 0.034	0.617 ± 0.027	0.619 ± 0.029	0.764
Choroid plexus volume (CPV) (cm^3^)	2.37 ± 0.78	2.03 ± 0.36	1.76 ± 0.48	< 0.001^*^
CPV/TIV ^*^ 10^3^	1.76 ± 0.61	1.51 ± 0.29	1.31 ± 0.35	< 0.001^*^
Enlarged perivascular space (EPVS) volume (cm^3^)	2.54 ± 0.68	2.24 ± 0.33	2.03 ± 0.43	< 0.001^*^
EPVS/TIV ^*^ 10^3^	1.89 ± 0.46	1.66 ± 0.26	1.51 ± 0.32	< 0.001^*^
ALPS	1.48 ± 0.12	1.57 ± 0.12	1.65 ± 0.12	< 0.001^*^

Data are represented as Mean ± SD.

^*^P < 0.001.

TSD, tinnitus with sleep disturbance; TNSD, tinnitus with no sleep disturbance; HCs, healthy controls; TIV, total intracranial volume; CPV, choroid plexus volume; EPVS, enlarged perivascular space; ALPS, analysis along the perivascular space.

### 3.3 Glymphatic function analysis

**Figure 2:**
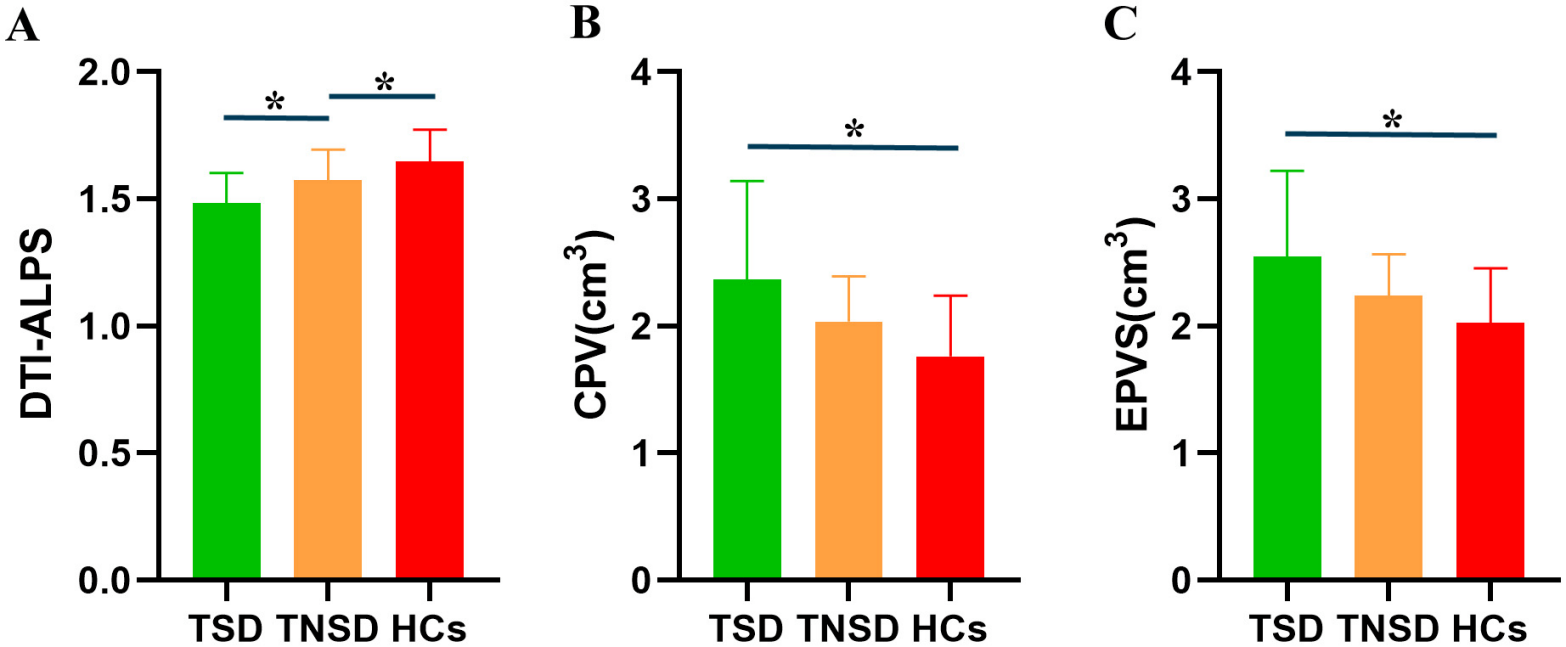
Differences of the glymphatic system function among TSD, TNSD and HCs. **(A)** TSD group showed significantly lower DTI-ALPS values than the TNSD (*p* = 0.018) and HCs group (*p* < 0.0001); TNSD group revealed significantly lower DTI-ALPS values than the HCs group (*p* = 0.044). **(B)** TSD group exhibited significantly higher CPV values than the HCs group (*p* < 0.0001). **(C)** TSD group exhibited significantly higher EPVS values than the HCs group (*p* < 0.0001). *Represents the significant differences between groups.

Using ANOVA, it showed there were significant differences of CPV, EPVS, and DTI-ALPS index among three groups (Figure 2). *Post-hoc* analysis indicated that the TSD group exhibited significantly higher CPV values than the HCs group (*p* < 0.0001) and significantly higher EPVS values than the HCs group (*p* < 0.0001). TSD group showed higher CPV and EPVS values than TNSD group but not significant (*p* > 0.05). Furthermore, the TSD group showed significantly lower DTI-ALPS values than the TNSD (*p* = 0.018) and HCs group (*p* < 0.0001). Additionally, the TNSD group revealed significantly lower DTI-ALPS values than the HCs group (*p* = 0.044). Detailed MRI indices of the participants are shown in Table 2.

### 3.4 Correlation analysis

**Figure 3:**
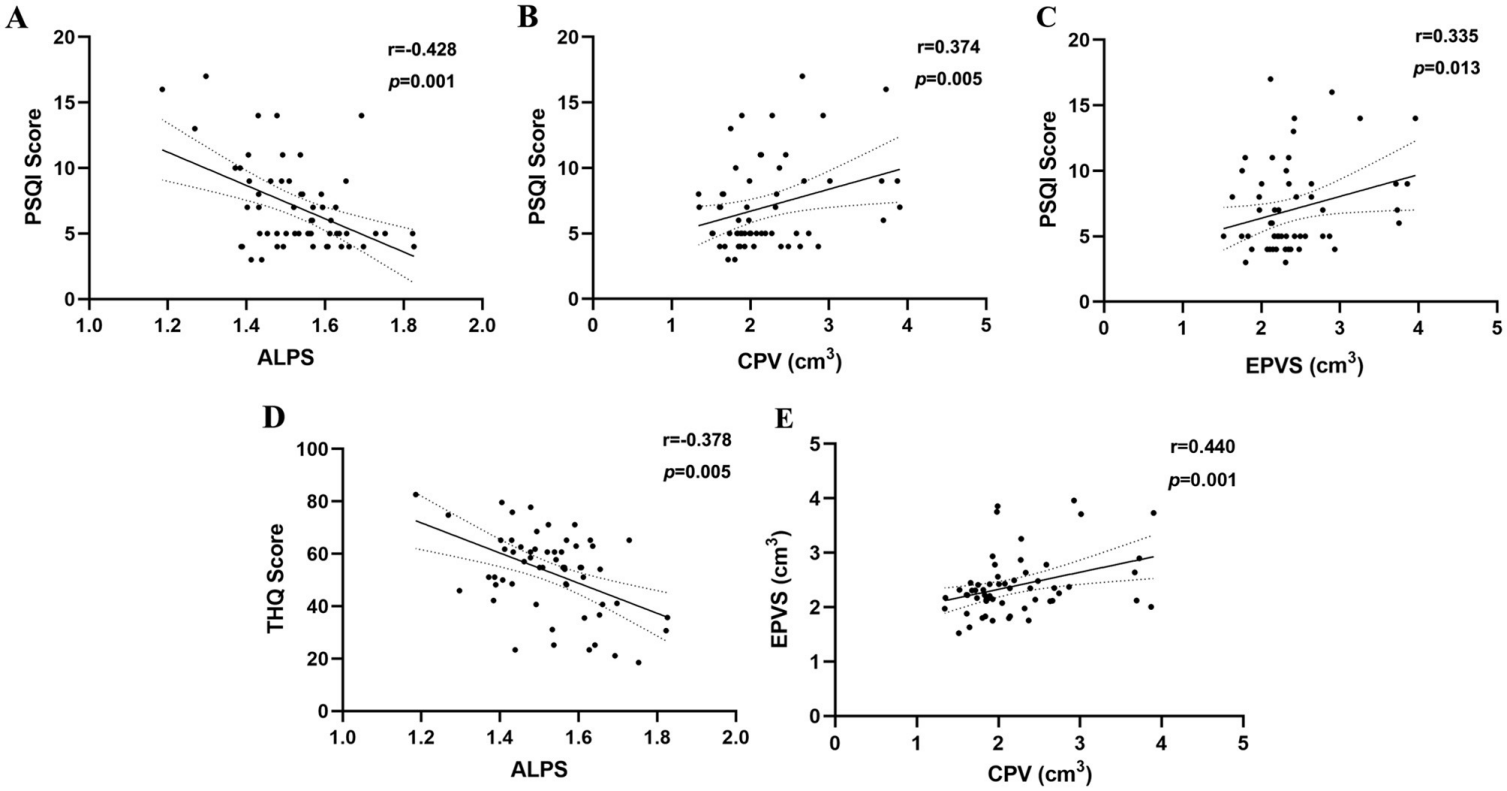
Correlation analyses between glymphatic system function and clinical variables. **(A)** In chronic tinnitus patients, the decreased DTI-ALPS index was negatively associated with the PSQI scores (*r* = −0.428, *p* = 0.001). **(B)** The increased CPV values were positively correlated with the PSQI scores (*r* = 0.374, *p* = 0.005). **(C)** The increased EPVS values were positively correlated with the PSQI scores (*r* = 0.335, *p* = 0.013). **(D)** The decreased ALPS values were negatively associated with the THQ scores (*r* = −0.378, *p* = 0.005). **(E)** The increased CPV values were positively correlated with the EPVS values (*r* = 0.440, *p* = 0.001).

Among all participants, age, gender, education, and average hearing thresholds were controlled as covariates to eliminate these variables as potential confounds. In chronic tinnitus patients, the decreased DTI-ALPS index was negatively associated with the PSQI scores (*r* = −0.428, *p* = 0.001) (Figure 3A). Moreover, the increased CPV and EPVS values were positively correlated with the PSQI scores (*r* = 0.374, *p* = 0.005; *r* = 0.335, *p* = 0.013; respectively) (Figures 3B, C). Furthermore, reduced ALPS values were negatively associated with the THQ scores (*r* = −0.378, *p* = 0.005) (Figure 3D). In addition, the increased CPV values were positively correlated with the EPVS values (*r* = 0.440, *p* = 0.001) (Figure 3E). However, no other MRI indices of glymphatic function were associated with other tinnitus clinical characteristics (all *p*>0.05). No significant correlations survived after Bonferroni correction.

## 4 Discussion

The current study explored for the first time to detect glymphatic dysfunction related to sleep disturbance in chronic tinnitus using multimodal MRI approaches. Our analyses non-invasively generated exploratory insights into the functional status of the glymphatic system, offering potentially relevant information for future clinical investigations. These metrics could be useful in assessing the role of the glymphatic system in chronic tinnitus patients with sleep disturbance.

In this study, we used multimodal MRI images to comprehensively evaluate the glymphatic system function in chronic tinnitus, including segmentation of CPV and EPVS utilizing T1w and calculation of ALPS index using DTI. The choroid plexus, regarded as a unique neuro-immunological interface, not only produces CSF, but also integrates signals from the CNS parenchyma with signals from circulating immune cells, and selectively recruits peripheral leukocytes to the CNS parenchyma (34). In Alzheimer's disease, increased CPV was associated with greater Aβ plaque formation and poorer cognitive function (16). EPVS volume is a major indicator of the increase in periarterial space, representing the inflow of CSF into the brain parenchyma (21). The ALPS index, which measures the spatial diffusion around the deep medullary vein, mainly reflects the outflow of CSF/ISF (22). Taoka et al. reported positive correlation between reduced DTI-ALPS index and cognitive dysfunction in Alzheimer's disease for the first time (18). We compared these indices in tinnitus groups and observed major differences suggesting that the glymphatic system is significantly impaired in individuals with chronic tinnitus. Moreover, the tinnitus glymphatic dysfunctions are correlated with sleep disruption and tinnitus distress. The correlations are at most moderate, but may also considered to be weak (35).

Chronic tinnitus patients showed higher CPV than HCs for reasons that are poorly understood. The choroid plexus not only plays a pivotal role in CSF production but is also essential for regulating the transfer of immune cells from the brain parenchyma into CSF (36). The mechanisms responsible for the tinnitus-related increase in CPV are unclear, but others have reported that CPV was associated with greater in Alzheimer's disease than HCs and was correlated with Aβ deposition and poorer cognitive function (16). Similarly, the volume of EPVS was significantly higher in tinnitus patients compared to HCs suggesting that the inflow of CSF into the brain parenchyma is impaired. AQP4, which is polarized in astrocytes, plays a significant role in volume regulation; inflammation can increase its expression which could lead to drainage disorders manifested as EPVS visible on MRI (37). The glymphatic system's role is to clear metabolic waste and interstitial solutes from the brain parenchyma, including the CSF tau protein (38). Our prior study found that chronic tinnitus patients revealed decreased DTI-ALPS index, which was correlated with specific neuropsychological tests (24). Thus, DTI-ALPS has been pivotal in detecting glymphatic system function and underscoring the potential value as a biological indicator of neuropathological conditions.

Nonetheless, the above three indicators based on diffusion and structural MRI cannot directly reflect dynamics aspects of the glymphatic system (20, 39, 40). Kiviniemi et al. suggested that low frequency (< 0.1 Hz) resting-state functional MRI (fMRI) blood-oxygen-level-dependent (BOLD) signals are linked to CSF dynamics and glymphatic function (41). The resting-state global BOLD (gBOLD) activity and associated physiological modulations are speculated to represent highly coordinated neural and physiological processes closely linked to glymphatic clearance (42, 43). Therefore, the gBOLD-CSF coupling may serve as a marker for evaluating the glymphatic function, and was linked to Aβ and tau in Alzheimer's disease, Parkinson's disease, as well as aging (44–48). The relationship between gBOLD-CSF coupling and glymphatic function in chronic tinnitus with sleep disturbance requires to be further investigated in future study.

Furthermore, the glymphatic system is primarily active during slow-wave sleep (49). Aberrant glymphatic function is associated with sleep disturbance in Alzheimer's and Parkinson's disease (50) as well as in our chronic tinnitus patients. Whether glymphatic dysfunction is a cause, consequence or bystander in these diseases remains to be determined. In our study, only lower DTI-ALPS values were detected in TSD group than TNSD group, which was associated with sleep disturbance. Increasing evidences have indicated that DTI-ALPS is a promising alternative indicator for evaluating the glymphatic function and has been widely used in various neurodegenerative diseases. Prior studies have suggested that glymphatic system impairment contributes to or is a result of sleep disorder or other sleep-related diseases using DTI-ALPS (13, 51, 52). Therefore, it raises the potential for DTI-ALPS as a potential biomarker for sleep disorder which will be an important question for future study. Further longitudinal studies with a larger sample of tinnitus patients will be acquired to determine whether similar associations exist in chronic tinnitus accompanied with sleep disturbance.

Several limitations should be acknowledged. First, the sample size was relatively small and it is based on cross-sectional analyses that make it difficult to establish a strong cause and effect relationship. Thus, long term longitudinal studies are acquired to investigate the changes of glymphatic function in tinnitus patients. Second, chronic tinnitus patients are often accompanied with mild to severe hearing loss. Future studies should include participants with hearing loss to determine if the correlation between the degree of hearing loss and glymphatic dysfunction in chronic tinnitus. Moreover, due to the lack of a group with sleep disturbances without tinnitus, it cannot be ruled out that the findings are associated with sleep disturbances alone irrespective of chronic tinnitus. In addition, although no correlations could pass such a stringent standard after Bonferroni correction (53), we believe that our research is still meaningful to provide some enlightenments for future study in this field. Finally, DTI-ALPS index provides a single measure of glymphatic function in each hemisphere. While reductions in the DTI-ALPS index have been reproducibly linked to multiple conditions associated with glymphatic impairment, it is an indirect measure, true confirmation of direct glymphatic contributions is difficult (20). Therefore, future glymphatic imaging studies of tinnitus would benefit from inclusion of more invasive methods to confirm the aforementioned findings.

## 5 Conclusions

In summary, this study provides preliminary evidence for reduced glymphatic function in chronic tinnitus patients compared to normal controls, which was correlated with sleep disruption, by using multimodal MRI approaches. Glymphatic dysfunction could represent a potential target for further investigation in developing therapies for chronic tinnitus patients with sleep disturbance.

## Data Availability

The original contributions presented in the study are included in the article/supplementary material, further inquiries can be directed to the corresponding author/s.
